# Electrochemical sensors for anticancer drugs used in the targeted therapy of chronic myeloid leukaemia

**DOI:** 10.5599/admet.2825

**Published:** 2025-07-18

**Authors:** Totka Dodevska

**Affiliations:** Department of Organic Chemistry and Inorganic Chemistry, University of Food Technologies, Plovdiv, Bulgaria

**Keywords:** Imatinib, dasatinib, nilotinib, bosutinib, ponatinib, asciminib, tyrosine kinase inhibitors, clinical analysis, therapeutic drug monitoring, electroanalysis

## Abstract

**Background and purpose:**

Treatment of chronic myeloid leukaemia includes targeted therapy with tyrosine kinase inhibitors (TKIs): imatinib, dasatinib, nilotinib, bosutinib, ponatinib, and asciminib. This review aims to prove that electrochemical sensors provide a reliable alternative to the conventional analytical methods for highly sensitive and cost-effective assay of TKIs in pharmaceutical formulations and biofluids. These platforms have significant advantages in fast detection and portability because they could be designed as miniaturized hand-held devices suitable for real-time point-of-care analysis, providing quick results for enabling personalized therapeutic drug monitoring.

**Experimental approach:**

The paper covers recent developments in substrate materials, various electrode designs, the advantages, and limitations of sensors for TKIs, encompassing both basic and applied research.

**Key results:**

This is a pioneering study that provides a general review on emerging trends, technologies, and practical applications of electrochemical sensors for TKIs analysis. The article provides researchers with a clear introduction and concise guide to the design and application of electrochemical sensors in the clinical analysis of TKIs.

**Conclusion:**

The review is intended to serve as a valuable resource for researchers in navigating the latest developments in TKIs' electrochemical sensing platforms. The fast response, high sensitivities and satisfactory recoveries obtained in blood serum and urine samples show the potential for application of the proposed electroanalytical systems in clinical analysis and optimization of chemotherapeutic treatments.

## Introduction

Chronic myeloid leukaemia (CML) is a blood cancer that starts in the blood-forming myeloid cells (stem cells) in the bone marrow. CML is a clonal myeloproliferative disease characterized by a reciprocal translocation between chromosomes 9 and 22 in the hematopoietic stem cell. Translocation of the Abelson murine leukaemia (ABL1) gene located on chromosome 9 to the breakpoint cluster region (BCR) gene located on chromosome 22 results in a BCR-ABL1 fusion gene [1]. The changed chromosome 22 with the fusion gene on it is called the Philadelphia chromosome (Ph). The novel chimeric protein BCR-ABL1 is a constitutively active tyrosine kinase that induces uncontrolled proliferation, differentiation arrest, and survival of leukaemia stem cells [2-5].

At the end of the last century, the discovery of the pathophysiology of CML made it possible to design a novel class of targeted therapeutic drugs, opening a new era in anti-cancer therapy through small-molecule tyrosine kinase inhibitors (TKIs). TKIs are pharmacological agents that effectively suppress the enzymatic activity, disrupting the signal transduction pathways of protein kinases, particularly the BCR-ABL1 TK, which is the cause of the leukemic transformation of Ph+ hematopoietic stem cells. In 2020, Jabbour and Kantarjian concluded that the “targeted” approach changed dramatically the CML therapeutic landscape, improving the 10-year survival rate of CML patients from approximately 20 to 90 % [1].

To date, the Food and Drug Administration (FDA) and European Medicines Agency (EMA) have approved six TKIs for use in CML therapy (Table 1): imatinib (IMA), dasatinib (DAS), nilotinib (NIL), bosutinib (BOS), ponatinib (PON), and asciminib (ASC) [1,6,7].

**Table 1.** Basic information for TKIs approved for CML therapy

Drug	Information	Trade names
Imatinib (IMA)	Empirical formula: C_29_H_31_N_7_O; Molecular weight: 493.6 g/mol Name:4-[(4-methylpiperazin-1-yl)methyl]-N-[4-methyl-3-[(4-pyridin-3-ylpyrimidin-2- -yl)amino]phenyl]benzamide	Glivec, Gleevec
Dasatinib (DAS)	Empirical formula: C_22_H_26_ClN_7_O_2_S; Molecular weight: 488.0 g/mol Name:N-(2-chloro-6-methylphenyl)-2-[[6-[4-(2-hydroxyethyl)piperazin-1-yl]-2- -methylpyrimidin-4-yl]amino]-1,3-thiazole-5-carboxamide	Sprycel, Dasanix, Daslemia, Invista
Nilotinib (NIL)	Empirical formula: C_28_H_22_F_3_N_7_O; Molecular weight: 529.52 g/mol Name:4-Methyl-3-[[4-(3-pyridinyl)-2-pyrimidinyl]amino]-N-[5-(4-methyl-1H-imidazol-1- -yl)-3-(trifluoromethyl)phenyl]benzamide	Tasigna, Neonib, Nilonix, Nilotin
Bosutinib (BOS)	Empirical formula: C_26_H_29_Cl_2_N_5_O_3_; Molecular weight: 530.4 g/mol Name:4-(2,4-dichloro-5-methoxyanilino)-6-methoxy-7-[3-(4-methylpiperazin-1-yl)propoxy]quinoline-3-carbonitrile	Bosulif, Bonitar
Ponatinib (PON)	Empirical formula: C_29_H_27_F_3_N_6_O; Molecular weight: 532.6 g/mol Name:3-(2-imidazo[1,2-b]pyridazin-3-ylethynyl)-4-methyl-N-[4-[(4-methylpiperazin-1-yl)methyl]-3-(trifluoromethyl)phenyl]benzamide	Iclusig, Ponaxen, Ponatinix, Ponatigen
Asciminib (ASC)	Empirical formula: C_20_H_18_ClF_2_N_5_O_3_; Molecular weight: 449.8 g/mol Name:N-[4-[chloro(difluoro)methoxy]phenyl]-6-[(3*R*)-3-hydroxypyrrolidin-1-yl]-5-(1H-pyrazol-5-yl)pyridine-3-carboxamide	Scemblix, Ascimib, Ascentib

Imatinib, the standard-of-care treatment for most CML patients, is the first-generation TKI (1GTKI) that was initially released in 2001. DAS, NIL, and BOS are second-generation TKIs (2GTKIs) that are more potent than IMA and active against several IMA-resistant BCR-ABL1 mutants. The 2GTKIs lead to a faster and deeper molecular response without a survival benefit compared to IMA [8]. However, patients who develop the T315I gatekeeper mutation display resistance to all currently available 1G and 2G TKIs [9].

PON is a third-generation TKI, more potent than all other TKIs. Due to its promising effect, in 2012, PON was granted accelerated FDA approval for the treatment of CML patients with the BCR-ABL1 T315I mutation and for patients with CML resistant to two or more TKIs [10].

ASC is the newest third-generation TKI that works as an allosteric inhibitor of kinase activity. In 2021, following approval by the FDA, ASC was welcomed as a novel therapeutic option endorsed by the National Comprehensive Cancer Network (NCCN). ASC has a unique mechanism of action - specifically binds to the ABL Myristoyl Pocket (STAMP) of the kinase domain, locks BCR-ABL into an inactive conformation, inhibiting downstream signalling events [11-13]. ASC showed a superior efficacy compared with that of BOS, together with a favourable safety profile [3,11,14].

Olverembatinib (OLV) is a new potent BCR-ABL1 TKI with preclinical activity against T315I-mutated CML and early promising results [15,16]. OLV is currently approved and marketed in China for the treatment of adult patients with TKI-resistant chronic-phase CML (CML-CP) or accelerated-phase CML (CML-AP) harbouring the T315I mutation [17]. In early 2024, the National Comprehensive Cancer Network (NCCN) added OLV to their latest guidelines for the treatment of patients with CML [18].

Adverse effects, including gastrointestinal effects, cytopenias, and hepatic toxicity, are common in patients receiving most types of cancer therapy. Cardiotoxicity has been asserted as a notable effect of TKIs therapy. Cardiovascular events have been frequently reported in TKI-treated CML patients, and their pathogenesis is still only partially understood [19-22]. Recent clinical studies indicate that elevations in blood cholesterol and glucose levels were more frequent at patients receiving NIL vs IMA. These findings indicate that NIL treatment is associated with increased risks of diabetes and hyperlipidemia [23]. In 2024, Zhao *et al.* [24] conducted a pharmacovigilance study to evaluate the adverse effects of BCR-ABL1 TKIs in cancer patients using the FDA Adverse Event Reporting System (FAERS) database. The results demonstrated that adverse event reports differ among the five TKIs. According to this study, NIL showed stronger signals in arteriosclerosis, which confirms the previously commented results. Table 2 summarizes the currently available six TKIs and their most frequently reported severe side effects.

**Table 2.** Currently available six TKIs and their most frequently reported severe side effects

TKI generation	Drug	Toxicity profile	Ref.
First (1GTKI)	IMA	cardiogenic shock/left ventricular dysfunction; hepatotoxicity (including liver failure); hypothyroidism; renal toxicity; bullous dermatologic reactions	[25]
Second (2GTKI)	DAS	pulmonary arterial hypertension; fluid retention (including pleuropericardial effusion); myelosuppression (thrombocytopenia, anemia, neutropenia) and bleeding event	[26]
	NIL	vascular pro-atherogenic properties causing arterial stenosis and vasospasm; arterial hypertension; myocardial ischemia or infarction; metabolic disorders including increased glucose, cholesterol and triglycerides levels; electrolyte abnormalities (hypophosphatemia, hypokalemia, hyperkalemia, hypocalcemia, and hyponatremia); myelosuppression; pancreatitis	[27]
	BOS	cardiac failure, left ventricular dysfunction, and cardiac ischemic events; hepatic toxicity - elevations in serum transaminases (alanine aminotransferase [ALT], aspartate aminotransferase [AST]); myelosuppression; gastrointestinal toxicity; renal toxicity	[28]
Third (3GTKI)	PON	arterial hypertension; increased incidence of: cerebrovascular and peripheral arterial occlusive events, venous thromboembolism, myocardial ischemia, infarction, angina pectoris; hepatotoxicity (including liver failure)	[29]
	ASC	arterial occlusive events; arthralgias; myelosuppression; pancreatitis	[30]

## Advances in the electrochemical determination of TKIs in biofluids and pharmaceutical formulations

Effective treatment is crucial to prevent rapid phase transfer in patients with CML [31]. Monitoring the concentration of anticancer drugs in biological fluids and pharmaceutical formulations is of paramount importance [32,33]. Quantitative analysis of drug formulations plays a crucial role in pharmaceutical quality control and regulatory compliance. On the other hand, selective determination of drugs in biological fluids is required for enabling personalized therapeutic drug monitoring (TDM). TDM is the clinical practice of measuring specific drugs in biological samples (plasma, blood) at designated intervals. TDM refers to the individualization of treatment by adapting drug dose to improve efficacy, manage side effects, and reduce toxicity [34]. In addition, the quantitative and qualitative analysis of drugs and their metabolites is extensively applied in pharmacokinetic studies.

In 2024, Chen *et al.* [35] presented a high-quality review paper that provides a comprehensive summary and comparison of pretreatment methods and analytical techniques specifically used for TKIs in biological samples since 2017. The article highlights the latest techniques, including gas chromatography (GC), high-performance liquid chromatography (HPLC), newly developed nanoprobes-based biosensing techniques, supercritical fluid chromatography (SFC) procedures, and high-resolution mass spectrometry (HRMS) methods. Authors concluded that nonconventional sensing methodologies, such as electrochemical methods, meet the requirements of TDM testing at the patient's bedside or in medical practices.

Electrochemical sensors attracted significant interest due to their high accuracy, extremely low sample volume, short analysis time, reduced solvent consumption, low cost, simple operation, and miniaturization capability [36-41]. A thorough review of recent publications on TKIs' electrochemical sensing systems suggests that voltammetric techniques such as differential pulse voltammetry (DPV), square wave voltammetry (SWV), and adsorptive striping voltammetry (AdsSV) have been frequently used due to their fast response and excellent performance (high selectivity, high accuracy, and good reproducibility). This review is organized in sections based on the type of electrode modification for signal amplification, highlighting the most promising platforms that enable personalized TDM in field-use electroanalytical systems.

### Electrochemical sensors based on unmodified electrodes

Cyclic voltammograms of DAS on glassy carbon electrode (GCE) [42] and disposable pencil graphite electrode [43], recorded in supporting electrolytes with different pH values, showed that DAS undergoes irreversible, pH-dependent oxidation. The oxidation peak reached maximum current value at pH ~3.0. Considering the results presented in these works, the oxidation of DAS involves the thiazole moiety. The overall transfer of two electrons and two protons from the sulphur atom of the thiazole ring leads to the formation of sulfoxide in the presence of water.

For the first time, in 2004, Hammam and co-workers studied the electrochemical behaviour of IMA in Britton-Robinson buffers of pH 2 to 11 at a hanging mercury drop electrode (HMDE) [44]. The drug was found to exhibit strong adsorption onto the electrode surface over the pH values 6 and 7. The cyclic voltammograms showed a single irreversible cathodic peak, attributed to the reduction of the C=O double bond of the molecule. IMA was determined quantitatively by HMDE with an accumulation step, with satisfactory results in pharmaceutical formulation and spiked human serum samples.

Boron-doped diamond electrode (BDDE) is a relatively new carbon-based sensing material useful for detecting organic compounds, including pharmaceuticals. BDDE have the widest potential window in aqueous solutions (ca 3.5 V) among all electrode materials, low and stable background current, and resistance to fouling. The electrochemical oxidation mechanism of IMA at BDDE platform was reported in 2016 by Brycht *et al.* [45]. It was established that the oxidation process (two electrons and two protons) occurs on the piperazine ring to form a quaternary Schiff base. Six years later, for the first time, Koszelska *et al.* [46] have investigated the electrochemical behaviour of PON on a BDDE. In supporting electrolyte HCl (pH 1.3), a well-defined oxidation peak was observed at a potential value of 1.35 V *vs.* Ag/AgCl. Authors reported that the electrochemical oxidation of PON is an irreversible process controlled by diffusion.

In a research paper published in 2020, the redox behaviour of NIL on a GCE in 0.1 M H_2_SO_4_, both in the absence and presence of the anionic surfactant sodium lauryl sulphate (SLS), was evaluated using different voltammetric methods [47]. According to this study, the negatively charged SLS monomers were attracted to the positively charged amine moiety on the pyrimidin-phenylamine structure through electrostatic interaction. Thus, a greater number of NIL molecules reached the GCE surface, leading to enhanced peak current on AdSSWV. The authors reported that NIL can be determined in a concentration range of 0.02 to 2 μM (limit of detection (LOD) = 6.33 nM) in electrolyte 0.1 M H_2_SO_4_ containing 0.2 μM SLS.

Table 3 summarizes the basic operational parameters of the as-referred unmodified electrodes.

**Table 3.** Electrochemical sensing platforms based on unmodified electrode materials.

Analyte	Electrode	Electrolyte	Technique	Potential, V*	Linear range, nM	LOD, nM	LOQ, nM	Ref.
DAS	GCE	Acetate buffer, pH 3.4	DPV	0.92	200 to 2,000	130	430	[42]
	PGE	B-R buffer, pH 3.0	AdsSWV	0.936	9.2 to 1,000	2.8	9.2	[43]
IMA	HMDE	B-R buffer, pH 6.0	SWAdCS	-1.14	0.9 to 30	0.26	0.87	[44]
	BDDE	B-R buffer, pH 2.0	DPV	1.0	30 to 250	6.3	21	[45]
PON	BDDE	HCl, pH 2.0	SWV	1.35	1,000 to 20,000	250	-	[46]
NIL	GCE	0.1 M H_2_SO_4_, pH 1.0	AdsSWV	1.099	20 to 2,000	6.33	10.2	[47]

*Reference electrode Ag/AgCl, 3 M KCl; Abbreviations: GCE - glassy carbon electrode; PGE - pencil graphite electrode; HMDE - hanging mercury drop electrode; BDDE - boron-doped diamond electrode; DPV - differential pulse voltammetry; AdsSWV - adsorptive striping square wave voltammetry; SWAdCS - square wave adsorptive cathodic stripping voltammetry; SWV - square wave voltammetry; B-R buffer - Britton-Robinson buffer; LOQ - limit of quantification

### Electrochemical sensors based on carbon nanomaterials

Disposable screen-printed carbon electrodes (SPCEs) provide attractive opportunities for the development of miniaturized cost-effective, robust, and quick sensing devices. High precision attained in the manufacturing processes provides high sensor-to-sensor repeatability and reliable analysis [48]. SPCEs have the potential for point-of-care (POC) applications needed for personalized health care as they can be used for the analysis of a single droplet of sample, thus allowing on-site analysis directly at the patient’s location [49]. In particular, SPCEs are becoming an essential tool in the development of efficient, simple, and reliable electroanalytical tools for monitoring the real-time concentration of anticancer drugs administered to patients in order to minimize the side effects of chemotherapy [50,51].

Generally, electrode modification enhances the analytical performance and applications. The modified SPCEs exhibited better electron transfer, improved catalytic properties, and an enhanced electroactive area compared to the conventional SPCEs. Appropriate nanosized materials used within the carbon ink or placed onto the electrode surface entail increased sensitivity, selectivity, and stability of sensors.

Carbon nanotubes (CNTs) have a large specific area, excellent conductivity, and accelerate the rates of electrochemical reactions. They can present in different shapes and geometries, namely single-walled and multi-walled carbon nanotubes (SWCNTs and MWCNTs). SWCNTs comprise a cylindrical graphene sheet of nanoscale diameter, while MWCNTs are composed of several concentric, coaxial, rolled-up graphene sheets. These materials have outstanding electrocatalytic activity in the redox behaviour of various substances and the capability to restrict surface fouling. CNTs-modified SPCEs possess a remarkable active surface area, chemical inertness, and low charge-transfer resistance in both aqueous and non-aqueous media, making them suitable for numerous applications [52,53].

In 2023, Hassanpour *et al.* [54] evaluated various types of SPCEs as different working electrodes for DAS detection, including those based on bare carbon modified with CNTs, SWCNTs, graphene, or graphene oxide. According to the CV results, among the different types of modified SPCEs, the single-walled carbon nanotube-modified electrode showed the highest anodic peak currents for DAS determination in Britton-Robinson buffer solution (pH 5.0). The measurement of DAS using the SWV technique demonstrates that SWCNTs/SPCEs can be utilized as an analytical tool for quantifying drugs in commercial pharmaceutical tablets.

For direct IMA determination, Rodríguez *et al.* [55] tested different types of SPEs as working electrodes, including electrodes based on carbon, modified carbon nanotube inks, gold nanoparticles, and platinum. Data suggested that the platinum electrode had no signal in both electrochemical techniques used, CV and SWV. However, independently of the applied technique, authors observed that the MWCNTs-COOH/SPCE offered higher peak intensity. The different electric charge between the MWCNT-COOH (negative charge due to the loss of the carboxylic group's proton) and the positive charge of IMA at pH 7.0 could explain the behaviour of IMA over the MWCNTs-COOH/SPCE and the high sensitivity of this platform. The sensing electrode combined with the square wave adsorptive stripping voltammetry (SWAdSV) technique exhibits a linear current response in the concentration range from 50 to 912 nM, LOD of 16 nM and LOQ of 55 nM, respecttively. The developed electrochemical platform is practically useful for the fast, direct determination of IMA in urine without any complex pre-treatment (just electrode electrochemical activation and urine dilution with a 20 mM phosphate buffer solution). Authors reported that the urine samples were collected from three drug-free volunteers and different CML patients with Gleevec treatment. The method offers a valuable alternative, reducing costs and saving time, while maintaining accuracy.

Compared to CNTs, graphene (GR) and its derivatives are widely used in a variety of electrochemical sensors due to their robust mechanical and electrical properties, flexibility, large surface area and the ability to control these properties through chemical functionalization. Graphene oxide (GO), reduced graphene oxide (rGO), functionalized and doped GO-based nanomaterials are emerging as promising modifiers for broad applications in electrochemical sensing. Despite the remarkable electrochemical behaviour of graphene-based electrodes, GR, GO, and rGO tend to aggregate, which causes deterioration of their electrochemical response. The modification with various compounds can tune their inherent properties, preventing the aggregation. In their recent work, Chen *et al.* [56] demonstrated that the complementary actions of rGO and chitosan raise the overall effectiveness of IMA sensing. The chitosan/rGO/GCE sensor’s effective operation in real sample analysis highlights its potential for application in clinical studies.

### Electrochemical sensors based on metal and metal oxide nanoparticles

Innovative strategies in sensor design are being developed to improve selectivity, sensitivity, and reproducibility. Nanosized materials are the most commonly used modifiers for their high surface area to volume ratios, high electron transfer efficiency, as well as excellent catalytic behavior. Advanced sensors based on metal nanoparticles (MNPs), metal oxide nanoparticles (MOxNPs), and nanocomposites have been developed with potential use in electroanalytical devices for rapid sensing of these TKIs.

For example, a nanocomposite film composed of Fe_3_O_4_@MWCNTs and polyacrylonitrile nanofibers (PANNFs) was reported for the determination of IMA [57]. The developed platform, Fe_3_O_4_@MWCNTs@PANNFs/CPE, offers good stability and a fast and sensitive response to IMA. The recovery value of IMA in spiked urine samples varied from 94 to 104 % suggesting the reliability of the proposed method for the determination of the target drug without significant matrix effects.

The utilization of biosynthesized nanomaterials is an alternative and promising tool to build sensing platforms without using hazardous reagents and is environmentally friendly at a low cost. A few review articles emphasize the significance of biosynthesized NPs in the field of electrochemical sensing [58-60]. These papers focus on recent achievements in electroanalysis using nanomaterials obtained through eco-friendly routes, primarily employing plant extracts. Actually, bioactive compounds in plant tissues act simultaneously as reducing, capping and functionalizing agents, enabling the NPs synthesis procedure to be completed in a single step. Literature survey revealed that the bio-assisted synthesis of metal and metal oxide NPs also played a role in the construction of electrochemical sensors for TKIs [61,62].

Wu *et al.* [62] used leaf extract of *Lycoris longituba* as a reducing agent for the one-step synthesis of reduced graphene oxide-Ag nanocomposites. The green synthesized rGO/Ag material was applied for surface modification of GCE and successfully used for electrochemical detection of IMA. Unfortunately, the authors do not provide information about the stability of the developed catalyst, so we cannot assess its practical effectiveness.

Korgaonkar *et al.* [61] have presented a novel approach by employing *Averrhoa bilimbi* leaf extract as a green reducing agent for the preparation of ZnO NPs. The biosynthesised ZnO NPs were mixed with functionalized MWCNT to prepare ZnO@f-MWCNT nanocomposite as an electrode material for electrochemical sensing of DAS. The developed sensor was used to determine DAS in fortified biological samples (human urine) and pharmaceutical formulations (DAS tablet marketed as “Invista 50”) by the proposed SWV method, showing high accuracy and precision. The authors reported only a 3.03 % decrease in the initial response of the peak current of DAS when the electrode material was kept at 4 °C for 30 days. These findings suggest that the modified electrode exhibits good stability and holds promising potential for pharmaceutical quality control and clinical studies.

For the fabrication of an electrode for the electrochemical detection of IMA, Baladi *et al.* [63] utilized graphitic carbon nitride g-C_3_N_4_. A new composite consisting of g-C_3_N_4_ and biosynthesized TbFeO_3_ (extract of *Diospyros kaki L*.) was used to modify GCE for the electroanalysis of IMA. The fabricated sensor demonstrated excellent detection limit (0.6 nM), repeatability, and acceptable accuracy in blood sample measurements.

Recently, Naderi *et al.* [64] used *Callicarpa maingayi* leaf extract as a reducing agent to reduce graphene oxide by a one-step method. A novel nanocomposite was developed by combining rGO sheets, CNTs, and Fe_3_O_4_ nanoparticles. Quantitative measurements of IMA at the surface of the modified electrode Fe_3_O_4_-(rGO&CNT)/CPE were performed by using CV. The proposed detection system has shown promising results in urine and blood plasma samples. It was observed that the data from electrochemical analysis show consistency with those obtained by UV-Vis spectroscopy, with acceptable recoveries.

Despite the advantageous properties of green synthesized nanomaterials, the major limitation for their widespread use consists in the fact that several parameters such as temperature, pH, extract purity and secondary metabolites, ratio precursor/extract solution are closely related to the physicochemical properties of NPs (size, crystal structure, surface morphology, agglomeration state), which can impact their electrical conductivity and electrocatalytic properties. Hence, efficient biosynthesis procedures must be validated on a case-by-case basis.

### Electrochemical sensors based on metal-organic frameworks and covalent-organic frameworks

Metal-organic frameworks (MOFs) are a relatively new family of inorganic-organic hybrid supramolecular materials that have attracted considerable attention in recent years. MOFs, known as coordination polymer networks or porous coordination polymers [65] are highly crystalline materials with multidimensional network structures formed by self-assembly of metal ions/clusters (inorganic metal-containing nodes) and organic ligands [66,67]. These materials possess unique inherent properties, including ultrahigh specific surface area, tunable intra-framework functionality, adjustable chemical properties, exposed metal sites, and ease of preparation. However, low intrinsic electronic conductivity and poor chemical stability in the aqueous medium of most MOFs concern their application in electrochemical sensing. Designing MOF-based nanocomposites with conductive materials, such as metal nanoparticles, metal oxide nanoparticles, graphene, carbon nanotubes, and carbon nanofibers, is an effective strategy to mitigate these drawbacks [65, 67-70]. For example, the electrochemical behaviour of CPE modified by a binary mixture of Ta/Cd MOF, which was electrochemically decorated with AuNPs, has been investigated in the DPV measurement of IMA [71].

Four research groups reported IMA electrochemical sensors based on CuMOF (Cu_3_(BTC)_2_ (BTC = 1,3,5-benzenetricarboxylate), square-pore MOF also known as HKUST-1 [72-75]. The authors refer to the results previously published by Abbasi *et al.* [76] on the absorption ability of CuMOF to IMA. Data clearly show that the IMA adsorption is governed by the host-guest interaction (the active empty cavity has a preferred IMB affinity through hydrogen bonding).

Jalal and coworkers reported the fabrication of an electrochemical sensor based on the *in situ* growth of framework HKUST-1 on conductive graphene oxide nanoribbons (GONRs)-modified glassy carbon electrodes [72]. A year later, Pour *et al.* [73] used MWCNTs as materials with high electrical conductivity to enhance the electron transfer capability of a glassy carbon electrode modified by the same CuMOFs. Both nanocomposites act as excellent modifiers for the preconcentration and detection of IMA in human blood serum and urine. The analytical results obtained with these sensors in terms of linear dynamic range and limit of detection are too close. In 2023, Xu *et al.* [74] developed a novel efficient sensor based on properties of acetylene black (AB) and CuMOF structures. Compared to the above-mentioned sensors for IMA determination, the prepared sensing platform CuMOF-AB/GCE exhibited a linear range in the nanomolar range and a generally lower LOD (1.7 nM). The accuracy of IMA quantitative determination in patient serum samples was compared with LC-MS and the results suggest the acceptable precision of the proposed method (RSD from 1.87 to 6.45 %).

**Figure 1.**
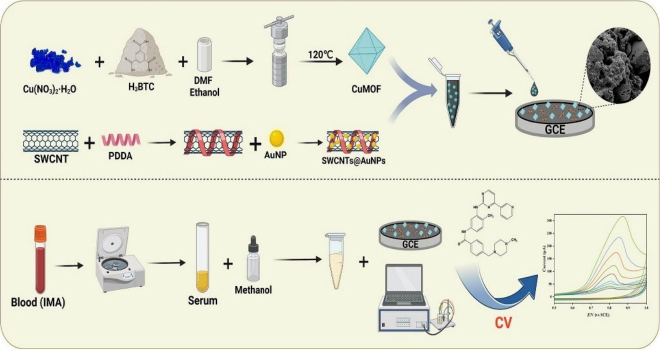
Schematic of a simple electrochemical sensor based on CuMOF-SWCNTs@AuNPs for detecting IMA in human blood serum [75] (CC BY 4.0 Attribution)

In 2024, a novel CuMOF-SWCNTs@AuNPs/GCE sensor for IMA was presented [75]. Positively charged poly(diallyldimethylammonium chloride) (PDDA) was used as a cross-linking agent to overcome the inherent van der Waals forces among pristine SWCNTs and further increase the loading ability of AuNPs (Figure 1). Authors reported linear response in the range from 0.05 to 20.0 μM and a detection limit of 5.2 nM. Overall, CuMOF-SWCNTs@AuNPs demonstrated promising candidates for electrochemical sensing, showing their potential application in therapeutic drug monitoring.

Habibi *et al.* [77] utilized the cavity of zeolitic imidazolate framework (ZIF-8) to encapsulate graphene quantum dots (GQDs) as well as CuFe_2_O_4_ magnetic nanoparticles to fabricate a new hybrid electrocatalytic material for electroanalysis of IMA. The developed sensor shows excellent performance for the trace detection of IMA with high sensitivity, low LOD (1.2 nM), and favorable accuracy. It is noteworthy that, after 28 days of storage (with electrochemical signals measured daily), the presented electrocatalyst retains up to 98.12 % of its initial activity. The fabrication methodology is reliable, as sensor-to-sensor reproducibility was investigated by measuring the current responses of eight identical CuFe_2_O_4_/ZIF-8@GQDs/GCE electrodes prepared independently using the same procedure. The results showed that the responses produced by different electrodes had excellent reproducibility, with an RSD of 2.23 %.

### Electrochemical sensors based on molecularly imprinted polymers

Molecularly imprinted polymers (MIPs) are chemically and mechanically stable artificial molecular recognition polymeric materials. Electrochemical sensors based on MIP as a functional component exhibit a unique capability for analyte recognition and excellent detection capability [78]. Remarkable properties of MIPs, such as cost-effectiveness and promising selectivity, make them attractive materials for catalysis applications.

For example, in 2024, Yıldız *et al.* [79] used molecular imprinting as a new strategy to create an extremely sensitive electrochemical method for determining DAS in human plasma and pharmaceutical tablets. Briefly, molecular imprinting involves the creation of specific recognition sites within a polymer matrix that can selectively bind to the target molecule (DAS). This is achieved via self-assembly of functional monomers around the template molecule (DAS), followed by the polymerization process that solidifies the structure. The authors employed surface-initiated photopolymerization as one of the most suitable methods for directly patterning MIPs on the transducer surface. Its main advantage is the tight control over film thickness, often at the nano-scale level [80]. Then, the template molecules were removed, leaving behind specific cavities that are structurally complementary to the template. For designing conductive MIP-based recognition elements, Yıldız and coworkers [79] used carbon nanofibers (CNFs) - promising biocompatible materials with conductivity and high specific strength that support efficient electron transport. The developed sensor platform demonstrated the ability to detect extremely low concentrations of DAS - the dynamic linear range was determined to be 10^-14^ to 10^-13^ M. Outstanding data for limits of detection and quantification are reported. The LOD and LOQ were determined in the femtomolar range: LOD = 1.76×10^-15^ M, and LOQ = 5.89×10^-15^ M. The relative standard deviation (RSD) values in tablet and commercial serum samples were found to be 1.96 and 1.36 %, respectively, indicating that the proposed method has excellent sensitivity. The authors concluded that the developed sensor could be used stably for up to 3 days. To examine the reusability of this sensing platform, the rebinding-removal cycle was repeated ten times using the same sensor. According to the data presented, the sensor was stable and maintained its performance around 93.41 % at the end of 10 cycles.

Although there is a genuine market need for such highly sensitive analytical devices, MIP-based technology has remained mostly in the academic field. In general, MIP-based electrochemical sensors have limitations, including mechanical compliance issues and sensor instability, which should be addressed in future research.

### Electrochemical sensors based on ionic liquids

**Figure 2.**
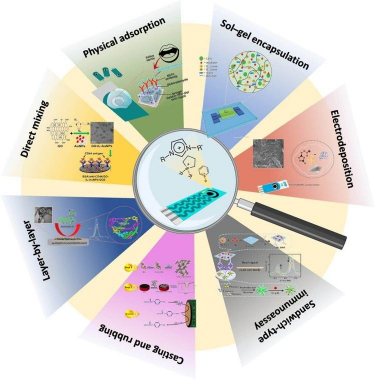
Scheme of different methods used for the fabrication of ionic liquids (ILs) electrochemical sensors [82] (CC BY 4.0 Attribution)

Another trend regarding electrode modifiers for electrochemical sensors concerns the combination of nanocomposites and ionic liquids (ILs) [81]. ILs are liquid electrolytes composed entirely of ions. Typically, IL has a bulky organic cation and a charge-delocalized anion. It has a low melting point (below 100 °C) and is typically in a liquid state. There are various methods for fabricating ILs-based sensors/biosensors, including direct mixing, physical adsorption, casting and rubbing, electrodeposition, sol-gel encapsulation, layer-by-layer assembly, and a sandwich-type architecture (Figure 2).

Because of their adjustable physical and chemical features, in recent years, more focus has been paid to ILs as effective modifiers for improving the sensing performance of electrodes. The use of ILs in electroanalytical applications is determined by their intrinsic properties. These materials possess many attractive characteristics such as good electrical conductivity, nonvolatility, hydrophobicity, wide electrochemical window, chemical stability, and high flexibility of the construction. They were used to prepare the electrode surface with a well-defined structure and enhanced detection sensitivity [81,82]. A variety of electrodes for TKIs electrochemical sensing have been fabricated based on the use of ILs.

For example, a graphite paste electrode amplified with a Fe_3_O_4_-SWCNTs nanocomposite and ionic liquid 1-hexyl-3-methylimidazolium tetrafluoroborate (mim-BF_4_^-^) was proposed as a new strategy for a highly sensitive electrochemical assay of DAS [83]. As the authors pointed out, the prepared electrode showed a LOD value of 0.7 nM by the SWV method, which is remarkable compared to those of previously reported modified electrodes. The sensor was also tested in tablets and a dextrose saline spike sample, yielding good results (recovery: 99.58 to 103.6%).

**Figure 3.**
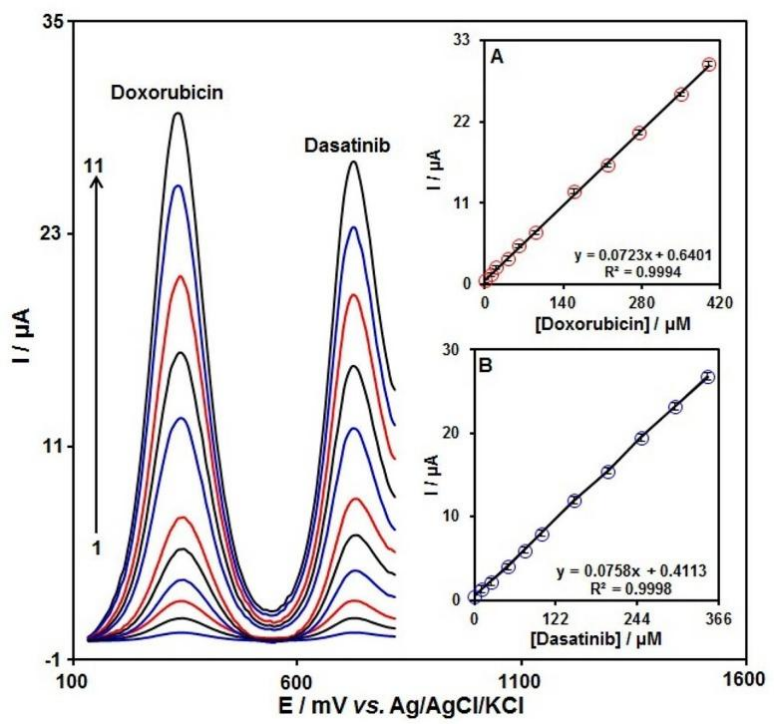
DPVs of the Fe_3_O_4_@MoS_2_/rGO/ILCPE in 0.1 M PBS at pH equal to 7.0 containing different concentrations of DOX and DAS. Notably, curves 1-11 correspond to 1.0 + 0.2, 10.0 + 1.0, 20.0 + 25.0, 40.0 + 50.0, 60.0 + 75.0, 90.0.0 + 100.0, 160.0 + 150.0, 220.0 + 200.0, 275.0 + 200.0, 350.0 + 300.0, and 400.0 + 350.0 μM of DOX and DAS, respectively. Inset A shows the *I*p plot vs DOX concentration. Inset B shows the Ip plot vs DAS concentration [84] (CC BY 4.0 Attribution)

Tajik *et al.* [84] in their recent research, synthesized a highly-sensitive electrocatalyst by combining Fe_3_O_4_@MoS_2_/rGO nanocomposite with IL and graphite powder, denoted as Fe_3_O_4_@MoS_2_/rGO/ILCPE for voltammetric detection of DAS in the presence of an anthracycline drug doxorubicin (DOX, trade name Adriamycin). According to previous studies, a significant synergy between DOX and DAS in inhibiting cancer cell proliferation has been demonstrated in various types of cancer cells [85,86]. The combination of DOX and DAS decreases the viability of stromal cancer stem cells, thereby contributing to the treatment of patients with metastatic soft tissue sarcoma, breast, prostate, and colon cancers. The modified electrode Fe_3_O_4_@MoS_2_/rGO/ILCPE exhibited high electrocatalytic activity toward the oxidation of DOX and DAS (Figure 3), with two well-resolved peaks on the differential pulse voltammogram (at 350 and 730 V *vs.* Ag/AgCl, corresponding to the oxidation of DOX and DAS, respectively). To ensure its suitability for practical applications, the electrode was tested in real samples (injection, tablet, and river water specimens) with acceptable recovery. However, the authors have not presented data on the simultaneous determination of DOX and DAS in an actual biological sample. Direct mixing was used for the fabrication of both IL-based sensors [83,84].

In another interesting work, Hatamluyi and Es'haghi [87] fabricated a novel sensor based on microextraction and electrochemical principles, simultaneously. Authors have created a layer-by-layer sensing architecture based on dendrimer and ionic liquid-supported reduced graphene oxide. The proposed sensing platform is a disposable tool for the separation, extraction, preconcentration and quantification of IMA. Briefly, IMA was pre-concentrated by dendrimer and rGO in the pores of the hollow fiber walls and further electrochemically detected through PGE modified by dendrimer/rGO nanocomposite. The results showed that the sensor can detect trace levels of IMA in urine and serum samples, with no cross-reactivity.

### Other types of electrochemical sensing platforms

In 2025, for the first time, Genc and co-workers [88] reported successful electrochemical quantification of asciminib in biological samples. The authors provided an electrochemical sensing platform based on Cr_2_AlC MAX phase/GCE. The MAX phases are layered hexagonal crystal structures that bridge the gap between ceramics and metals and consequently have attracted great attention in recent years with their unique properties. The data indicated that the Cr_2_AlC MAX phase would exhibit good electrocatalytic activity. The results, along with the Nernst equation calculations, revealed that the ASC oxidation process followed a one-proton and one-electron process. The corresponding RSD of 11 consecutive DPV measurements of 1.0 μM ASC in 0.1 M Britton-Robinson buffer (pH 2.0) was 1.1 %. Moreover, reproducibility, as examined by preparing 10 similar modified electrodes, was 2.0 %. The developed sensing platform has been substantially applied to reliably determine ASC in serum, urine, and drug formulation (tablet) with satisfactory recovery rates ranging from 100.4 to 102.5 %. These findings underscore the reliability of the presented electrochemical methodology for ASC quantification in various real samples.

Yıldır *et al.* [89] demonstrated the synergetic signal amplification towards NIL oxidation of two different materials - CoS and nitrogen-doped amorphous porous carbon. The analytical platform based on the fabricated modified electrode (CoS@NAPC/GCE) was used for the quantification of NIL in capsules and urine [89].

An electrochemical biosensor is a self-contained, integrated, analytical device in which a biological recognition element (enzymes, antibodies, antigens, peptides, DNA, aptamers or living cells) is retained in direct spatial contact with an electrochemical transducer. The biosensor converts the interaction between the biomolecule and the target analyte into an electric current (or potential). The choice of biomaterial and its integration into the sensor platform design are key factors that determine the analytical performance. In this regard, the novel biomolecule−nanomaterials hybrid systems have excellent prospects.

**Figure 4.**
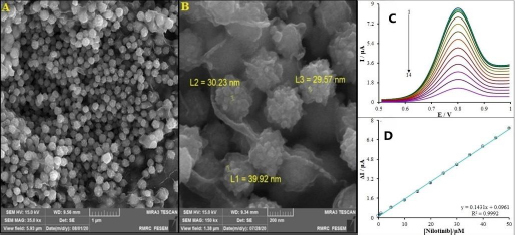
(A) FESEM image; (B) high-resolution FESEM image of In^3+^/NiO RLHNSs; (C) Voltammograms of ds-DNA/In^3+^/NiO RLHNSs/CPE for different concentrations of NIL in ABS (0.1M, pH 4.8), from top to bottom (1-14), 0.0, 0.01, 0.5, 1.0, 5.0, 10.0, 15.0, 20.0, 25.0, 30.0, 35.0, 40.0, 45.0 and 50.0 μM; (D) dependence of the net oxidation guanine current (different between guanine current in the absence and presence of NIL) *vs.* concentration of NIL [91] (CC BY-NC-ND 4.0 Attribution).

Deoxyribonucleic acid (DNA)-based biosensors have emerged as powerful tools for rapid, sensitive and specific detection and quantification of various analytes at low concentrations [90]. The nucleic acid recognition layer has been used to detect modifications in the DNA structure as a result of intercalations with DNA-binding molecules, including drugs. Moarefdoust *et al.* [91] introduced a DNA biosensor for NIL, based on a carbon paste electrode modified with three-dimensional (3D) raspberry-like In^3+^/NiO hierarchical nano-structures (In^3+^/NiO RLHNS) (Figure 4). The electrochemical detection of NIL is based on observing modifications in the oxidation signal of guanine. Consequently, the authors have explored the interaction mechanisms of ds-DNA with NIL using UV-Vis spectroscopy, voltammetry, computational docking, and viscosity measurements. The team reported a LOD of 0.62 nM. This new DNA biosensor (ds-DNA/In^3+^/NiO RLHNSs/CPE) was utilized to detect NIL in human blood serum and urine samples using the standard addition method, showing recovery rates between 98.0 and 101.3 %. Lifetime of the sensing platform is 70 measurements with current loss less than 8 %. The biosensor demonstrated high reproducibility, with an RSD of 2.28 %, and retained 97.6 % of its activity after 30 days of storage in 0.1 M PBS, highlighting its long-term stability. This innovative approach presents a promising avenue for the development of highly sensitive and selective bioelectroanalytical systems for NIL assay, making them crucial for various applications, including monitoring, diagnostics, and research.

In another study published in 2024 [92], the interaction mechanism between PON and dsDNA was investigated for the first time using electrochemical and computational techniques, such as the semi-empirical method PM7 and density functional theory (DFT). The obtained data will contribute to a better understanding of the mechanism of interaction of this tyrosine kinase inhibitor with nucleic acids. Based on the voltammetric results obtained at physiological pH, the authors have concluded that PON interacts with both dGua and dAdo residues in double-stranded DNA (dsDNA) molecules. The results obtained with both PM7 and DFT methods indicate that in all four sites, the PON molecule forms stable complexes with dsDNA, regardless of the DNA model used.

In conclusion, the comparative Table 4 provides a valuable resource for researchers navigating the latest developments in TKIs electrochemical sensing platforms. The fast response, high sensitivity and satisfactory recoveries obtained in blood serum and urine samples showed the potential for application of the proposed electroanalytical systems in clinical analysis and optimization of chemotherapeutic treatments.

**Table 4.** Electrochemical sensors based on modified electrode materials for quantitative detection of TKIs

Sensing electrode	Analyte	Technique	Linear range, μM	LOD, nM (LOQ, nM)	Real sample	Stability, % (Period)	Ref.
HF-PGE	IMA	DPV	0.01 to 200	7.39 (24.6)	Serum, urine	−	[87]
rGO-Ag/GCE	IMA	DPV	0.010 to 280	1.1 (−)	Serum	−	[62]
HKUST-1/GONRs/GCE	IMA	DPV	0.04 to 80	6 (−)	Serum, urine	95 (9 days)	[72]
CuMOFs-MWCNTs/GCE	IMA	DPV	0.01 to 220	4.1 (−)	Serum, urine, tablet	96 (1 month)	[73]
CuMOF-AB/GCE	IMA	CV	0.0025 to 6	1.7 (−)	Serum	94.65 (7 days)	[74]
CuMOF-SWCNTs@AuNPs/GCE	IMA	CV	0.05 to 20	5.2 (−)	Serum	82.3 (15 days)	[75]
CHIT/rGO/GCE	IMA	DPV	1 to 300	7.3 (−)	Serum, urine	97 (1 month)	[56]
MWCNT-COOH/SPCE	IMA	SWV	0.050 to 0.912	7 (23 )	Urine	−	[55]
CuFe_2_O_4_/ZIF-8@GQDs	IMA	DPV	0.02 to 94.2	1.2 (4.1)	Serum, tablet	98.12 (28 days)	[77]
Fe_3_O_4_@MWCNTs@PANNFs/CPE	IMA	DPV	0.002 to 0.850	0.4 (−)	Urine	96 (5 weeks)	[57]
TbFeO_3_/g-C_3_N_4_/GCE	IMAT	DPV	0.002 to 100	0.6 (−)	Plasma	−	[63]
PGE	DAS	AdsSWV	0.0092 to 1	2.8 (9.2)	Urine	−	[43]
MATyr-CNF@MIP/GCE	DAS	DPV	10^-5^ to 10^-4^	1.76** (5.89**)	Serum, tablet	96.8 (3 days)	[79]
SWCNT/SPCE	DAS	SWV	0.1 to 100	60(190)	Tablet	−	[54]
Fe_3_O_4_-SWCNTs/mim-BF_4_^−^/PE	DAS	SWV	0.001 to 220	0.7 (−)	Tablet	92 (60 days)	[83]
ZnO@*f-*MWCNT/GCE	DAS	DPV	0.03 to 82.65	480(157)	Tablet, urine	97 (30 days)	[61]
		SWV	0.01 to 122.45	52(16)			
ds-DNA/In^3+^/NiO RLHNSs/CPE	NIL	DPV	0.01 to 50	0.62 (−)	Serum, urine	97.6 (30 days)	[91]
GCE in the presence of SLS	NIL	AdsSWV	0.02 to 2	6.33 (10.2)	Serum, urine	−	[47]
BDDE	PON	SWV	1 to 20	250 (−)	Urine	−	[46]
Cr_2_AlC MAX phase/GCE	ASC	DPV	1 to 10	212(698)	Tablet, urine, serum	10 days	[88]
CoS@NAPC/GCE	NIL	DPV	59.7 to 1570 & 1570 to 11200*	11.8* (39.3*)	Capsules, human urine	95.45 (5 days)	[89]

*ng mL^-1^

**fM

Abbreviations: HF-PGE - hollow fiber-pencil graphite electrode; rGO - reduced graphene oxide; HKUST - MOF type ([Cu_3_(H_2_O)_3_(BTC)_2_]n, BTC:1,3,5-benzenetricarboxylate); MWCNT - multi-walled carbon nanotubes; SWCNT - single-walled carbon nanotubes; AB - acetylene black; CHIT - chitosan; SPCE - screen-printed carbon electrode; mim-BF_4_^−^ − 1-hexyl-3-methylimidazolium tetrafluoroborate; PE - paste electrode; (*f*-MWCNT) - functionalized multi-walled carbon nanotubes; In^3+^/NiO RLHNSs - raspberry-like indium(III)/nickel oxide hierarchical nano-structures; CPE - carbon paste electrode; SLS - sodium lauryl sulfate; BDDE - boron-doped diamond electrode; Cr_2_AlC MAX phase - two-dimensional transition metal carbide; NAPC - nitrogen-doped amorphous porous carbon.

### Electrochemical sensors for the simultaneous determination of TKIs and other chemotherapy drugs

Recently, new electroanalytical methods for multiplexed analysis are required to ensure fast and precise results. Multiplexed analysis reduces the time and total cost of analysis, requires sample volumes, and is less labor-intensive [93,94]. In this regard, the simultaneous detection of multiple analytes with different redox potentials using a single working electrode is feasible. Among the electrochemical techniques, SWV stands out for its advantages, including fast analysis, background discrimination, and increased sensitivity. Careful research has shown that only a few electrochemical sensors can simultaneously detect two TKIs, providing more comprehensive information for diagnostic purposes [95-98].

The combination of DOX and DAS can be effective for the treatment of breast, prostate, and colon cancers. According to several studies, this approach is promising for decreasing or inhibiting the migration, proliferation, cell metabolism, and invasion of cancer cells. Moreover, such therapy is also useful for minimizing drug resistance. Regarding the simultaneous detection of DOX and DAS using a single electrode, it should be pointed out that the experimental conditions may not be optimal for both analytes being measured. As an effective strategy, researchers modified the electrode surface to change the redox reaction kinetics. This approach results in high resolution of peaks, higher selectivity and sensitivity, hindering accurate determination of both analytes. Novel mesoporous bimetallic Pd@Pt core-shell nanoparticles supported on MWCNTs have been synthesized by Kalambate *et al.* [97] and employed as an electrochemical platform for individual as well as simultaneous determination of DOX and DAS. Due to the synergistic effect of these structures, which facilitate fast electron and mass transfer, the sensor showed an intrinsic advantage in highly sensitive electrochemical detection of both anticancer drugs. Their results have shown satisfactory recovery rates with good reproducibility, indicating potential applications of the proposed system in clinical analysis.

Alavi-Tabari *et al*. [96] described another amplification method for the simultaneous detection of DOX and DAS by using ZnO nanoparticles/1-butyl-3-methylimidazolium tetrafluoroborate modified carbon paste electrode. The ZnO-NPs/BMTFB/CPE exhibits an oxidation peak-separation potential of 0.3 V during the simultaneous analysis of DOX and DAS. The detection ability of the developed electrode was also demonstrated in commercially available pharmaceutical specimens, yielding satisfactory results and highlighting the sensor’s promising potential.

6-mercaptopurine (6-MP) and 6-thioguanine (6-TG) are chemotherapeutic drugs in the treatment of childhood acute lymphoblastic leukaemia [99]. For the first time, Karimi-Maleh *et al.* [100] have reported the simultaneous electrochemical determination of 6-MP, 6-TG, and DAS by incorporating Pt/MWCNTs and ionic liquid BMIHFP into the carbon paste matrix. A potential difference of 200 mV between 6-MP and 6-TG and 290 mV between 6-TG and DAS were detected, which is large enough to determine the above compounds individually and/or simultaneously with good outputs.

The comparison of sensors designed for the simultaneous detection of anticancer drugs is presented in Table 5.

**Table 5.** Electrochemical sensors for the simultaneous determination of anticancer drugs.

Sensing electrode	Analyte	Technique	Potential, V*	Linear range, μM	LOD, nM	Real sample	Stability, % (Period)	Ref.
Pd@Pt/MWCNTs/ /GCE	DAS	AdsSWV	0.792 V	0.038 to 9.88	6.72	Urine, serum	95.5 (30 days)	[97]
	DOX		0.468 V	0.044 to 8.580	0.86			
ZnO-NPs/ /BMTFB/CPE	DAS	SWV	1.0 V	1 to 1200	500	Injection, serum	93 (2 weeks)	[96]
	DOX		0.718 V	0.07 to 500	9			
Pt/MWCNTs-BMIHFP-CPE	DAS	SWV	0.95V	5 to 500	1,000	Urine	97.1 (45 days)	[100]
	6-TG		0.66 V	0.1 to 500	50			
	6-MP		0.46 V	0.05 to 550	9			

*Reference electrode Ag/AgCl, 3 M KCl; BMTFB - 1-butyl-3-methylimidazolium tetrafluoroborate; BMIHFP - 1-butyl-3-methylimidazolium hexafluorophosphate; DOX - doxorubicin; 6-TG - 6-thioguanine; 6-MP - 6-mercaptopurine.

## Challenges and future directions

Recently, electroanalysis has become a hot topic in the field of clinical analysis. Compared to other conventional methods, such as chromatography and mass spectrometry, the electrochemical sensing platforms are much more straightforward, cost-effective and easier to miniaturize, which makes them more suitable for point-of-care detection. Therefore, the development of novel, powerful electrochemical sensors is a driving force for dramatic change in point-of-care health monitoring. Such systems can be used for the assay of TKIs and optimization of chemotherapeutic treatment for patients with CML. The review highlights a variety of electroanalytical techniques (CV, DPV, SWV, and SWAdSV) that have been applied in the analysis of TKIs. The presented overview clearly shows that a lot of inspiring and promising electrochemical sensing platforms for TKIs have emerged over the last few years. Key innovations such as the use of novel nanomaterials, modified nanocomposites, MIPs, and ionic liquids have been emphasized for their critical roles in improving sensor analytical performance. Such rapid and reliable sensors are capable of revolutionizing chemotherapeutic monitoring by allowing the correct anticancer drug to be delivered at the right time in a patient-tailored dose. However, there are some challenges regarding the commercialization of such analytical devices that should be considered in future studies, for instance:

1. *Development of antifouling interface.* Electrode fouling/biofouling in real samples (biofluids) remains a critical issue during the determination of TKIs in blood serum and urine samples. This phenomenon refers to the accumulation of chemical species/biomolecules on the electrode surface that obstructs the electron transfer process of the analyte of interest. Thus, electrode fouling severely affects the analytical behaviour of the sensor in terms of sensitivity, LOD, and reproducibility. Research groups need to explore novel active anti-fouling electrode materials and new design strategies for sustainable improvement of the accuracy, reliability, and operational lifetime of electrochemical sensors for TKIs.
2. *Miniaturization and digitization of sensing platforms.* Owing to the breakthrough in novel nanosized electrode materials, sensors can be miniaturized without loss of analytical performance using scalable methods to create marketable products. Some of the proposed laboratory prototypes of electrochemical sensors for TKIs have the potential to be manufactured into compact, robust devices for real-time monitoring of drug levels in patients undergoing chemotherapy, offering valuable insights into personalized health care.
3. *Development of multianalyte sensors for simultaneous electrochemical quantification of TKIs.* Nowadays, the electrochemical sensors for multiple analyte detection have gathered significant attention in personalized medicine. The review showed that mainly single TKI sensing electrodes have been proposed. Design and development of sensors for simultaneous detection of two TKIs or TKI and blood markers such as creatinine (a marker for kidney function assessment) or bilirubin (low levels are associated with a greater risk of coronary heart disease and anaemia) are highly advantageous and desirable. Additionally, utilizing wearable technologies is a pathway to “electrochemical lab-on-a-chip” miniaturized devices. Engineering of smart wireless multiplexed sensor networks is also an important task.
4. *Integration of electrochemical sensors with artificial intelligence (AI) technology.* The incorporation of AI and machine learning (ML) algorithms with their capacity to facilitate sophisticated data processing will inevitably improve TKIs' monitoring. Algorithms using deep learning are effective for resolving peak overlap in voltammetric analysis when the target analytes have similar electrochemical behaviour. Full integration with big data platforms will contribute to rapid data collection and has the potential to generate predictive models supporting real-time analysis. Therefore, AI can assist sensor readout directly, accurately, and rapidly, which is important for on-site detection and decision-making.

## Abbreviations

AB: acetylene black
AdSSWV: adsorptive stripping square wave voltammetry
ASC: asciminib
BMIHFP: 1-butyl-3-methylimidazolium hexafluorophosphate
BMTFB: 1-butyl-3-methylimidazolium tetrafluoroborate
BOS: bosutinib
BTC: 1,3,5-benzenetricarboxylate
CHIT: chitosan
CML: chronic myeloid leukaemia
CNF: carbon nanofiber
DAS: dasatinib
DOX: doxorubicin
DPV: differential pulse voltammetry
GCE: glassy carbon electrode
GQDs: graphene quantum dots
GR: graphene
HF-PGE: hollow fiber-pencil graphite electrode
IMA: imatinib
IMAT: imatinib mesylate
MATyr: N-methacryloyl-L-tyrosine
mim-BF_4_^−^: 1-hexyl-3-methylimidazolium tetrafluoroborate
MIP: molecularly imprinted polymer
MOFs: metal-organic frameworks
6-MP: 6-mercaptopurine
NIL: nilotinib
PANNFs: polyacrylonitrile nanofibers
PGE: pencil graphite electrode
PON: ponatinib
rGO: reduced graphene oxide
SLS: sodium lauryl sulfate
SPCE: screen-printed carbon electrode
SWAdCS: square-wave adsorptive cathodic stripping voltammetry
SWAdSV: square wave adsorptive striping voltammetry
SWV: square wave voltammetry
6-TG: 6-thioguanine
TDM: therapeutic drug monitoring
TKIs: tyrosine kinase inhibitors
ZIF: zeolitic imidazolate framework
